# Supplementary material for: Distinctive Features of Orbital Adipose Tissue (OAT) in Graves’ Orbitopathy
Source: Int J Mol Sci. 2020 Nov 30;21(23):9145. doi: 10.3390/ijms21239145 (PMC7730568; doi:10.3390/ijms21239145)
Supplement: Supplementary file 1 [file ijms-21-09145-s001.zip › Supporting Information 2020.pdf]

Supporting Information (1 figure, 5 tables)

Figure S1. No changes of *FASN* transcripts expression in WAT, OAT-H/OAT-GO. Total RNA was isolated from OAT-H (n=4), OAT-GO (n=9) and WAT (n=6) *ex vivo* fat tissues. Transcripts of *FASN* was analysed as comparative QPCR (relative ratio to housekeeper gene *APRT*). Histograms = mean±SEM of all samples studied.

Table S1. DGE analysis of OAT-H (n=4) compared with WAT (n=5) by R/Bioconductor DESeq2 package. Data set in excel sheets with gene ID, Chromosome (Chr), gene name, baseMean, lfcSE, stat, pvalue and FDR-p were shown. **A**) Full data set sheet has interested genes sub-grouped for guidance including lipid metabolism, Wnt/Ca+ signalling, IGF, FGF signaling, TGFb/SMAD/BMP signalling, neurological activities, angiogenesis, forkhead transcriptional factors, complement system, development of WAT/BAT and other important cellular activities, in which the bold font indicated significantly up-regulated genes in OAT compared with WAT. **B**) A separate excel sheet showed data with criteria of $\log_2$fold Change of OAT-H versus WAT (>0.5 or <-0.5), and FDR-p (<0.05). Top 500 $\log_2$fold Change of up- or down-regulated genes (FDR-p<0.05) were highlighted in grey with top 10 up- and down-regulated genes in yellow.

Abbreviations: baseMean, normalised counts, averaged over all samples from both OAT-H and WAT; lfcSE, the lfcSE gives the standard error of the log2FoldChange; stat, used in Wald statistic as the log2FoldChange divided by lfcSE, which is compared to a standard Normal distribution to generate a two-tailed pvalue; pvalue, Wald test p-value as above; padj (FDR-p): the p-value adjusted for multiple testing with the Benjamini-Hochberg.

Table S2. Bio-functions analysis of OAT-H by IPA® using DGE data (FDR-p<0.05; $\log_2$ fold changes>0.5 or <-0.5). Bio-functions of OAT-H were analysed with overlap-

p value and z-score indicating inactivation (-) or activation compared with WAT. Down- or up-regulated gene set of OAT-H versus WAT with p value (-p) and z-score (-Z) was also analysed separately. The number (#) of genes involved in each function were listed and significant (overlap-p<0.01) activation or inactivation were measured with z-score >2 or <-2, respectively.

Table S3. Signaling pathways analysis of OAT-H DGE data (FDR-p<0.05; log_2fold changes>0.5 or <-0.5) using IPA®. Signaling pathways of OAT-H were analysed with overlap-p value and z-score indicating inactivation (-) or activation compared with WAT. Down- or up-regulated gene set of OAT-H versus WAT with overlap-p value (-p) and z-score (-z) was also analysed separately. The grey highlighted pathways are detected significant changes, overlap-p<0.01 and z-score >2 (activation) or <-2 (inactivation); bold font indicated the activated signaling pathways. Ratio is showing the mapping number of genes (listed in table) at the same pathway of the total number of genes from Ingenuity® Knowledge Base. Brief description IPA® report of signaling pathways of Sirtuin, Wnt/Ca+ and FGF were provided following the table content.

Table S4. Growth factors identified in OAT-H compared with WAT. DGE analysis of OAT-H compared with WAT, Log2fold changes and significant FDR-p<0.05 were shown with identified up- (bold font) or down- (in grey) regulated growth factors.

Table S5. Primer for QPCR used in this study with indicated exon (Ex) location and gene accession number for mRNA analysis.
