# Supplementary material for: Distinctive Features of Orbital Adipose Tissue (OAT) in Graves’ Orbitopathy
Source: Int J Mol Sci. 2020 Nov 30;21(23):9145. doi: 10.3390/ijms21239145 (PMC7730568; doi:10.3390/ijms21239145)
Supplement: Supplementary file 1 [file ijms-21-09145-s001.zip › Table S5.pdf]

Gene	Forward Primer	Reverse Primer
LEP (NM_000230)	AATGCATTGGGGAACCCT GT (Ex2)	AGGAGACTGACTGCGTGTGT (Ex3)
UCP1 (NM_021833)	GGGGCTTCAGCGGCAAA TCAG (Ex2)	TATAAGTCCCCGTGTAGCGA GGTT(Ex3)
LPL (NM_000237)	GAGATTTCTCTGTATGGA CC (Ex7)	CTGCAAATGAGACACTTTCTC (Ex9)
LIPE (NM_005357)	ACTCAGACCAGAAAGCCC TC (Ex8)	GGGTCAGGTTCTTGAGGGAA (Ex9)
SCD (NM_005063)	TGCCCACCACAAGTTTTC AG (Ex4)	CATCAGCAAGCCAGGTTTGT (Ex5)
MFN2 (NM_014874)	CGTCTATGAGCGTCTGAC CT (Ex17)	TTTCTTGTTCATGGCGGCAA (Ex18)
FASN (NM_004104)	ACCACCCGCTCGGCATG GCTATCT (Ex31/32)	CTTGTGGGCCGGGCAGAAG GTCTT (Ex33)
SLC27A6 (NM_014031)	TAAGGGGTTCTGCTGTCC TG (Ex4)	TCACTCCAAAACTGGCTTGC (Ex5)
APRT (NM_000485)	GCTGCGTGCTCATCCGAA AG (Ex3)	CCTTAAGCGAGGTCAGCTCC (Ex5)

Table S5. Primer for QPCR used in this study with indicated exon (Ex) location and gene access number for mRNA analysis.
