# Supplementary figures and images for: Distinctive Features of Orbital Adipose Tissue (OAT) in Graves’ Orbitopathy
Source: Int J Mol Sci. 2020 Nov 30;21(23):9145. doi: 10.3390/ijms21239145 (PMC7730568; doi:10.3390/ijms21239145)

**Figure S1**

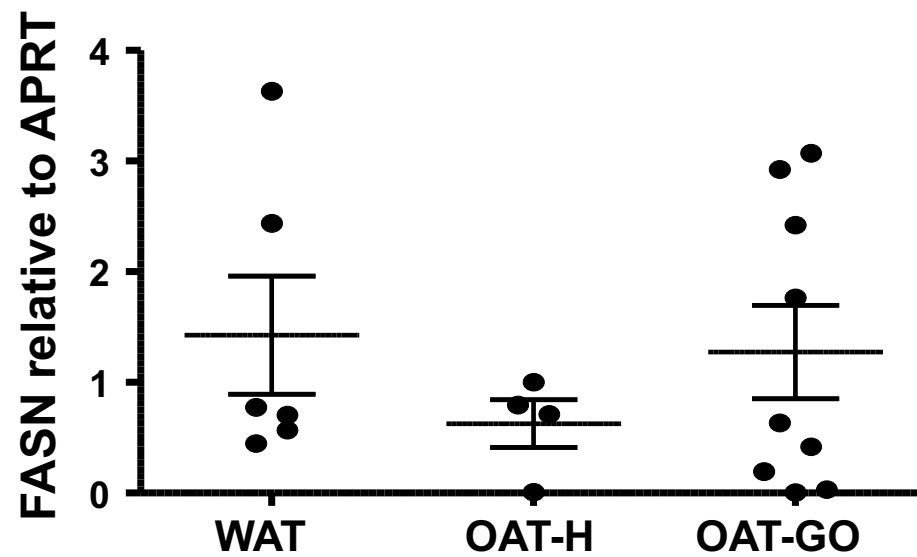

Supplement: Supplementary file 1 [file ijms-21-09145-s001.zip › Figure S1.pdf]
